# Quantifying spatial accessibility of general practitioners by applying a modified huff three-step floating catchment area (MH3SFCA) method

**DOI:** 10.1186/s12942-021-00263-3

**Published:** 2021-02-17

**Authors:** Julia Subal, Piret Paal, Jukka M. Krisp

**Affiliations:** 1 Applied Geoinformatics, University of Augsburg, Institute of Geography, Alter Postweg 118, 86159 Augsburg, Germany; 2 WHO Collaborating Centre, Institute for Nursing Science and Practice, Paracelsus Medical University, Strubergasse 21, 5020 Salzburg, Austria

**Keywords:** Spatial analysis, Spatial accessibility, General practitioners, Floating catchment area method, Oversupply, Undersupply, Health inequities

## Abstract

**Background:**

It is necessary to ensure sufficient healthcare. The use of current, precise and realistic methods to model spatial accessibility to healthcare and thus improved decision-making is helping this process. Generally, these methods—which include the family of floating catchment area (FCA) methods—incorporate a number of criteria that address topics like access, efficiency, budget, equity and the overall system utilization. How can we measure spatial accessibility? This paper investigates a sophisticated approach for quantifying the spatial accessibility of general practitioners. (GPs). Our objective is the investigation and application of a spatial accessibility index by an improved Huff three-step floating catchment area (MH3SFCA) method.

**Methods:**

We modify and implement the huff model three-step floating catchment area (MH3SFCA) method and exemplary calculation of the spatial accessibility indices for the test study area. The method is extended to incorporate a more realistic way to model the distance decay effect. To that end, instead of a binary approach, a continuous approach is employed. Therefore, each distance between a healthcare site and the population is incorporated individually. The study area includes Swabia and the city of Augsburg, Germany. The data for analysis is obtained from following data sources: (1) Acxiom Deutschland GmbH (2020) provided a test dataset for the locations of general practitioners (GPs); (2) OpenStreetMap (OSM) data is utilized for road networks; and (3) the Statistische Ämter des Bundes und der Länder (German official census 2011) provided a population distribution dataset stemming from the 2011 Census.

**Results:**

The spatial accessibility indices are distributed in an inhomogeneous as well as polycentric pattern for the general practitioners (GPs). Differences in spatial accessibility are found mainly between urban and rural areas. The transitions from lower to higher values of accessibility or vice versa in general are smooth rather than abrupt. The results indicate that the MH3SFCA method is suited for comparing the spatial accessibility of GPs in different regions. The results of the MH3SFCA method can be used to indicate over- and undersupplied areas. However, the absolute values of the indices do not inherently define accessibility to be too low or too high. Instead, the indices compare the spatial relationships between each supply and demand location. As a result, the higher the value of the accessibility indices, the higher the opportunities for the respective population locations. The result for the study area are exemplary as the test input data has a high uncertainty. Depending on the objective, it might be necessary to further analyze the results of the method.

**Conclusions:**

The application of the MH3SFCA method on small-scale data can provide an overview of accessibility for the whole study area. As many factors have to be taken into account, the outcomes are too complex for a direct and clear interpretation of why indices are low or high. The MH3SFCA method can be used to detect differences in accessibility on a small scale. In order to effectively detect over- or undersupply, further analysis must be conducted and/or different (legal) constraints must be applied. The methodology requires input data of high quality.

## Contributions to the literature

With this article, we contribute to the literature body of healthcare planning and document methodologies in measuring the availability of healthcare. Our focus is particularly on measurements for spatial accessibility, exemplary in the case of general practitioners. These findings contribute to gaps in current methodologies.

## Introduction

Especially in times of a growing as well as aging population and increasing prosperity on a global level, there is a simultaneously both constant and growing demand for healthcare in rural and urban settings [1]. The constitution of the World Health Organization (WHO) [2] states that "*[t]he enjoyment of the highest attainable standard of health is one of the fundamental rights of every human being without distinction of race, religion, political belief, economic or social condition*". In the United Nations’ (UN) 2030 Agenda for Sustainable Development [3], all of its members have committed themselves to the goal of achieving universal health coverage. This includes being able to access high-quality essential healthcare services. Particularly today, where every country has to handle the outbreak of the coronavirus disease 2019 (COVID-19), the topic of healthcare and thus access to healthcare is more important than ever. However, the goal of a world where every person has adequate access to healthcare is far from being met [4].

Germany represents a country with a well-established healthcare system, a high number of physicians and a generally good access to healthcare [5]. Nevertheless, there are ongoing challenges that the German healthcare system has to face. According to the Robert Koch-Institut [6], three big long-term issues can be identified: (1) the demographic shift, which results in an aging population, (2) the inequalities in health due to social differences and (3) rising numbers of non-communicative diseases (NCDs) like cardiovascular diseases, cancer or diabetes mellitus. With the current events due to the outbreak of COVID-19, issues associated with the virus can be added to this list. In order to overcome these issues, having universal access to healthcare services in urban as well as rural areas is crucial. The OECD State of Health report [7] provides a concise and policy-relevant overview of health and health systems in the European Economic Area. According to this report, the share of the population over age 65 is 21.2% in Germany. Especially poor diet, smoking and alcohol consumption are driving forces for morbidity and mortality which adds to the burden of NCDs. Germany has the highest levels of per capita health expenditures (11.2% of the gross domestic product) and the highest rates of hospital beds, doctors, and nurses per population in the EU. A dense network of healthcare professionals and hospitals ensure an overall high availability of care across Germany, albeit with lower availability in rural areas. Only half of the German population in the lowest income group have self-reported good health compared to 80% of those in the highest income group, which indicates socio-economic inequality. In 2020 German healthcare has demonstrated a great resistance in containing infectious disease, but it is not clear how the Covid-19 outbreak has affected the care continuum for those suffering from acute care needs, chronic conditions or mental-ill health. Again, in particular for people living in rural areas and for those from lower socio-economic status the risks of unmet care are estimated to be higher. In Germany, while the number of doctors has grown in stationary as well as outpatient care, the share of general practitioners (GPs) has decreased since 2000. In 2016, only 16.7% of doctors worked as GPs, which was 25% lower than the average share in the EU. Still, for the majority of the population, the closest GP is less than 1.5 km away. However, rural areas can face a potential shortage of doctors, which can lead to longer travel distances for patients. There is no gatekeeping system, which means that patients do not need referrals from GPs to visit medical specialists or enroll in hospitals. As the role of nurses as primary care provider is not recognized in German healthcare, all consultations are physician-led which comes with high consultation costs. Access to healthcare in Germany is good, but the provision of care and care transitions are fragmented, resulting in enormous healthcare expenditures. Covid-19 restraints on economic growth are expected to affect the healthcare sector. Hence, specific models for cross-sectional planning are needed. This paper investigates an improved floating catchment area (FCA) method as a tool to indicate over- and undersupplied areas in terms of access to GP provided healthcare services.

### Defining spatial access to healthcare

According to Morris et al. [8], accessibility can in short be defined as the relative ease by which certain locations, for example the workplace, shopping facilities or healthcare services, can be reached from other locations. With regard to healthcare, accessibility deals with all processes that are involved when residents are entering the healthcare delivery system [9]. This system includes among others GPs, medical specialists, such as oncologists or neurologists, and hospital facilities [10]. A renowned definition (see e.g. [11–16]) by Penchansky and Thomas [17] states that healthcare access is "*a concept representing the degree of fit between the clients and the system*". They suggest that healthcare access is a multilayered concept consisting of five areas. *Accommodation*, *affordability* and *acceptability* are considered to be aspatial areas concerned with the appropriateness, cost and compliance of healthcare services whereas *availability* and *accessibility* are spatial areas. Availability and accessibility can thus be referred to as *spatial accessibility* [18]. The availability of services can be described by the capacity of the services, for example, by the number of hospital beds or the number of available practitioners. The accessibility of services can be measured using the space between the demand and the healthcare supply. This space is influenced by the travel impedance between the demand and supply location [10, 17, 19].

Another frequently used definition of healthcare access (see e.g. [11, 16, 18–20]) was brought up by Khan [21] and Khan and Bhardwaj [22]. They state that healthcare access is (1) a dichotomous matter and (2) about the interaction between the individual and the healthcare system. First, Khan [21] and Khan and Bhardwaj [22] differentiate between the *spatial* and *aspatial* dimension of access. While spatial access stresses the role of spatial separation between supply and demand, aspatial access emphasizes nongeographic barriers such as language or ethnicity [23, 24]. Furthermore, Khan [21] and Khan and Bhardwaj [22] integrate the dimensions of *potential* and *realized* access. While potential access is concerned with the possible use of a service and can be derived using e.g. information about the population size and its demographics, realized or revealed access measures the actual use of healthcare services [11, 21, 23]. These four dimensions can be combined into potential spatial, realized spatial, potential aspatial and realized aspatial access. This paper’s method investigates the *potential spatial* access.

## Research aims and objectives

Considering the limited existence of resources such as the diminishing number of GPs in rural areas and the growing need for primary healthcare services, establishing comprehensive (spatial) access to healthcare services poses a challenge. Therefore, difficult decisions have to be made, for example about where healthcare facilities will be allocated [25]. As previous studies have shown, the better the access and the closer people are to healthcare services, the more likely it is that they will use these services. In other words: access and proximity are highly correlated with the use of healthcare services [26–30]. However, healthcare services are by nature distributed in a heterogeneous manner. Hence, access varies across space and leads to inequalities in healthcare acquisition. Therefore, difficult decision-making and planning is necessary to make access as ubiquitous as possible [24]. To that end, it is essential to be able to assess the current state of the healthcare supply structure [31].

Currently, the legal basis for decision making and planning of the statutory healthcare supply in Germany is the Bedarfsplanungs-Richtlinie (BPL-RL) [32]. Its purpose is to make sure that no areas are over- or undersupplied. In 2019, the BPL-RL was reformed. This reform allows for the consultation of Geographic Information Systems (GIS) when deciding upon admissions where the approval of a panel physician is necessary to ensure sufficient healthcare in a certain area. Due to this revision, the use of current, precise and realistic methods to assess spatial access to healthcare and thus improved decision-making is made possible [15, 18, 33, 34]. Generally, these methods—which include the family of floating catchment area methods—incorporate a number of criteria that address topics like access, efficiency, budget, equity and the overall system utilization [20, 35].

How can we measure spatial accessibility? Our aim is to demonstrate a sophisticated approach for quantifying the spatial accessibility of GPs, in particular the *Modified Huff Model three-step floating catchment area (MH3SFCA) method* and suggest further improvements for the method. As the name implies, the MH3SFCA method is part of the family of floating catchment area (FCA) methods.

## Related work and introduction to the method

Historically, the widespread and renowned 2SFCA method by Luo and Wang (2003) emerged from earlier versions of the FCA methods. These earlier versions were mainly used to quantify job accessibility (see e.g. [60–63]). They furthermore have similarities to kernel estimations (see e.g. [59]). In the earlier FCA methods, a catchment area (or kernel) is moved (“floats”) across the area of interest. The density of events within the catchment is then estimated to equal the density of events at the center of this catchment [19]. The 2SFCA method in particular is based on two preceding methods: the spatial decomposition method by Radke and Mu [65] and the advanced gravity-based method by Weibull [36].

Vo et al. [16] state that the FCA methods have undergone rapid progress over the last decade. Numerous improvements of the 2SFCA method have been proposed. Noteworthy at this point, as they all are related to the MH3SFCA method, are the Enhanced two-step floating catchment area (E2SFCA) method by Luo and Qi [41], the three-step FCA (3SFCA) method by Wan et al. [44], the Modified two-step floating catchment area (FCA) (M2SFCA) method by Delamater [20] and the Enhanced three-step FCA (E3SFCA) method by Luo [34, 46]. The E2SFCA method by Luo and Qi [41], which is based on studies by Guagliardo [18] and Alford et al. [64], aims to include the distance decay effect into the original 2SFCA method. It does so by incorporating different weights for different time zones. The 3SFCA method has been proposed by Wan et al. [44]. Its aim is to curb the aforementioned problem of demand overestimation occurring in the 2SFCA and E2SFCA methods [20]. To that end, Wan et al. [44] include the competition between health care services. In other words, the demand is influenced by the availability of health care service sites. Like the 3SFCA method, the Enhanced 3SFCA (E3SFCA) method proposed by Luo [34, 46] aims to overcome the problem of overestimation in the 2SFCA and E2SFCA methods. However, instead of weights based on travel time like in the 3SFCA method, Luo [34, 46] employs a modified Huff Model for this purpose. The M2SFCA method was proposed by Delamater [20] to overcome issues of both the E2SFCA and the 3SFCA method. Although the E3SFCA method was developed later than the M2SFCA method, the M2SFCA method provides a number of advantages over the E3SFCA method. Delamater [20] points out that the assumption that a ratio of demand to supply is optimal will lead to an overestimation of spatial accessibility. To provide a solution for this new kind of overestimation problem, Delamater [20] takes a suboptimal configuration of service locations into account by orienting the calculation of the 2SFCA method towards a modified version of the gravity model.

Despite the differences between the vast number of proposed FCA versions, there are a number of characteristics that all of them have in common. Firstly, all FCA methods are enhancements of the advanced gravity model. Therefore, they belong to the category of gravity-based spatial accessibility models [34, 36]. Like the gravity model, they incorporate information about supply (i.e. the healthcare services in the area of interest), demand (i.e. the population size in the area of interest) and distance to quantify spatial accessibility [20]. Secondly, these methods combine aspects of both regional availability (i.e. supply–demand-ratios) and regional accessibility. Hence, they are conceptually complete as they include both dimensions of spatial access [17, 18]. The basic approach of all FCA methods is the same. Using concepts stemming from geography, econometrics and applied physics, they are used to calculate an accessibility index in different geographical settings [16]. Moreover, all methods have three characteristics in common:

- They integrate the relevant supply and demand locations and use information from both;
- They quantify the relationship between supply and demand (availability);
- They quantify the spatial relationship between supply and demand (accessibility) while using distance in a way that is independent from administrative (or other fixed) borders [31].

The result of all FCA methods is an *accessibility index*. This index depends on the following criteria, whereby the index is rising with.

- A higher number of supply locations and a higher capacity,
- A lower amount of demand and
- An increasing proximity of supply to demand locations [31].

These criteria emerge from the way the FCA methods are calculated mathematically—in other words, the criteria can be derived through the variables used in the FCA methods. In contrast to indices that result from e.g. traditional gravity models, the index resulting from the FCA methods is made of container-based, well interpretable units [24]. These units are the opportunities per person, i.e. for example the number of physicians or beds per person [19, 20]. Another benefit of the FCA methods is that they incorporate the *distance decay effect*, however with varying accuracies. This effect describes the decreasing probability of residents to interact with a healthcare service the higher the distance between the residents and the service is [37]. Several studies have shown the distance decay effect to be persistent for different kinds of settings and healthcare services [23, 38, 39]. However, the intensity of the effect is varying due to the differing severities and urgency of health concerns. Mathematically, the distance decay effect is most commonly described using a (inverse-)power function, an exponential function, a Gaussian function or a logistical function [30, 31, 40]. Apart from the choice of a specific distance decay function, the distance decay effect can be modelled in various ways. The easiest way to model the distance decay effect is as a discrete variable. This is for instance done in the binary approach, where populations within a spatial unit or catchment have equal access to a service (value of the function equals 1) and no access at all outside the unit or catchment (value of the function equals 0) [24].

Another possibility to model the effect in a discrete way is by using multiple weights [24]. Using multiple subzones for the spatial unit or catchment, each subzone is assigned a certain weight. This weight is normally calculated by using a certain distance decay function (such as the Gaussian function). The respective weights are then used to weigh the accessibility for residents within each subzone. Residents in a subzone further away from a healthcare service therefore have less access to the service than residents in a closer subzone [41]. However, modeling the distance decay effect in a discrete way does not conform to natural conditions. Hence, various studies have attempted to improve the integration of the distance decay effect by modeling it in a continuous, gradual manner (while still using a threshold distance). An alternative strategy that integrates both the discrete and continuous approach is a hybrid approach [24], which is seldomly applied in the context of studies concerned with the FCA methods. In this paper, a recent FCA method is extended to incorporate a more realistic way to model the spatial distance decay effect. To that end, we use a continuous approach to distance decay in combination with the Gaussian function.

## Materials and methods

We conduct a case study with exemplary results. The study area is the administrative region Swabia, which is located within the federal state of Bavaria in Germany. Concerning the required data, a network based on OpenStreetMap (OSM) data is used. As this data is crowd-sourced it has varying quality and high uncertainties. For the creation of the network dataset, an appropriate transport mode needs to be selected. Despite a decline in car trips over the past years, the car is still the vehicle of choice in Germany for an average of above a third of the trips in cities [42]. In rural areas, the private car even constitutes the dominant traffic mode [43]. Hence, the network is configured in such a way that calculations use the transport mode by car. Additionally, the driving time (as opposed to the distance) is added to the network as the travel mode since most studies concerned with the FCA methods rather use the travel time than the travel distance (see e.g. [15, 19, 44]).

For the healthcare locations, a suitable dataset is provided by Acxiom Deutschland GmbH[45] for this study. The dataset comprises three different types of healthcare providers: (1) GPs, (2) dentists and (3) ophthalmologists. In this case study, we focus on the GPs. A dataset stemming from the 2011 census, provided by the Statistische Ämter des Bundes und der Länder,^1^ is utilized for the population’s distribution. This dataset contains the number of inhabitants in Germany for 100 by 100 m (i.e. hectare) grid cells. The dataset is originally prepared by aggregating the number of inhabitants on the address level (Kirchner et al. 2014). Whilst reporting our results we have consulted the Standards for QUality Improvement Reporting Excellence (SQUIRE 2.0).^2^

### The Modified Huff Model three-step floating catchment area method

Originally presented by Jörg et al. [31], the MH3SFCA method is largely based on two preceding FCA methods, namely the MH2SFCA method by Delamater [20] and the E2SFCA method by Luo [34, 46]. As such, the method incorporates the probability of interaction between a healthcare service site and a population location through integrating the Huff Model by Huff [47, 48]. By using this approach, the overestimation of demand—which occurs because the ability of alternative services to cover a part of this demand is ignored in many previous FCA methods—is avoided [20, 31, 41, 44]. In addition, a distance weight serves as a means to integrate absolute distances in addition to relative ones. The innovation of the MH3SFCA method in comparison to all previous FCA methods is the integration of a constant total demand. Therefore, in the MH3SFCA method, the demand of a population only decreases with an increasing distance to a service site if alternative service sites are available. Hence, the MH3SFCA method is highly sophisticated as it combines several advantages of previous traditional methods with advantages of more advanced methods. Especially the advantages of the MH3SFCA method over traditional methods are manifold. Compared to simple supply–demand-ratios, they include for example the independence of the results from a spatial unit, the consideration of relative distance differences within the maximum travel time and the consideration of supply competition [31].

**Fig. 1:**
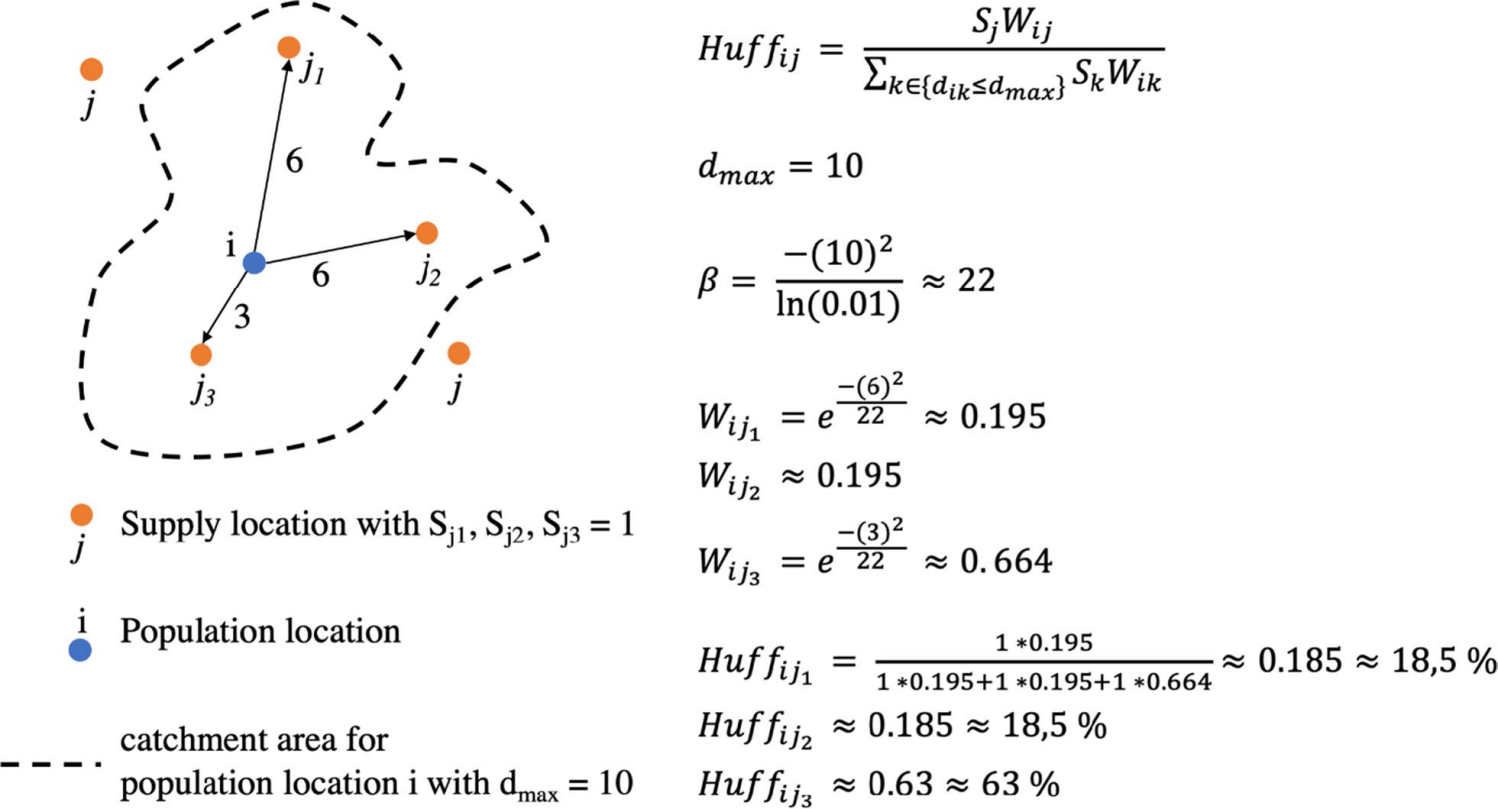
A visualization and example of the modified first step of the MH3SFCA method. Originating from the population location $i$, the probability of interaction $Huff_{ij}$ is calculated for the three pairings with the supply locations $j_{1}$, $j_{2}$ and $j_{3}$. The supply locations are located within the travel time catchment ($d_{max} = 10$) of $i$. The coefficient of friction is calculated for the chosen $d_{max}$ by rearranging the Gaussian function $f\left( d \right) = e^{{\frac{{ - d^{2} }}{\beta }}}$

We modify the original method by Jörg et al. [31] with the goal of incorporating a more realistic way to model the distance decay effect [34, 44, 46]. Jörg et al. [31] calculated Gaussian weights for four subzones in total. To that end, they chose the median of the travel time of the respective subzone to calculate the weights. For example, a subzone reaching from 0 to 5 min would be assigned the Gaussian weight for d = 2.5. This approach is similar to that of the E2SFCA method by Luo and Qi [41]. However, this approach assumes accessibility in terms of travel time to be equal within each subzone. Instead of defining subzones within the catchment areas, we employed a continuous approach. To that end, the calculation of the Gaussian weights is adapted so that instead of assigning weights according to different subzones, each pair of population and healthcare location are assigned an individual weight. The calculation of these weights is shown in Fig. 1. It should be noted at this point that the choice between using subzones or using a continuous approach depends heavily on the size of the study area, the available hardware and the overall objective. For example, if the goal is to assess accessibility to healthcare within a country, the use of subzones is sufficient. However, if the goal is to achieve a reliable assessment of accessibility in a (very) small area, for example to help plan the location of new healthcare sites, a continuous approach might be more sensible.

Initially, catchment areas were calculated for each population and health care location. For this, the travel times between each pair of demand and supply locations were required. To obtain these travel times, an Origin–Destination Cost Matrix function was applied. In order to calculate the travel times, the function employs a so-called multiple-origin, multiple-destination algorithm. This algorithm is based on the well-known Dijkstra algorithm. Aside from the employed network’s speed limits, general driving conventions such as one-way streets are considered. The travel times resulting from the function are the shortest-path travel times. Additionally, the travel times are only included for paths that are within the specified travel time threshold. This threshold is, besides the so-called coefficient of friction β, one of two critical parameters of the MH3SFCA method. Both should at best be based on empirical data or patient behavior [40, 41]. However, such information was not available for this study. Therefore, various studies were consulted to be able to make a reasonable choice for the threshold. A study by Lee [57] suggests that the acceptable travel time thresholds can vary in relation to the type of health care provider as well as to the condition of the infrastructure in the country. For example, to reach dental and psychiatric care in the USA, he deems 40 min to be appropriate. For primary care, he lowers the threshold to 30 min. Other studies employ travel times ranging from 20 to 60 min [31, 58]. Generally, most studies assume that for densely populated and developed (regarding the infrastructure) areas, 30 min are appropriate [19, 41, 57]. This value is determined as the longest acceptable travel time in a German study by Voigtländer and Deiters [55] as well. They state that within Germany, a travel time threshold of 30 min (albeit for using public transport) is reasonable and travel times above this value indicate a possible undersupply. To be able to (1) compare the resulting accessibility indices for all three types of providers, to (2) determine a value that would be acceptable for all three types and to (3) bring together the various suggestions, we chose 30 min as the travel time threshold.

The MH3SFCA method is then mathematically calculated as follows:

*Step 1*: The further away a service is from a population, the lower are the weight and accordingly the probability of interaction, $Huff_{ij }$. Following this premise, $Huff_{ij }$ is calculated by

$$Huff_{ij } = \frac{{S_{j} W_{ij} }}{{\mathop \sum \nolimits_{{k \in \left\{ {d_{ik} \le d_{max} } \right\}}}^{{}} S_{k} W_{ik} }}$$

where $S_{j}$ is the capacity (i.e. the number of practitioners) of a certain healthcare service site $j$. $d_{max}$ is the maximum travel time. $k$ is any healthcare service located within the catchment of $i$ ($d_{ik} \le d_{max}$). $W_{ij}$ and $W_{ik}$ are the corresponding, individual Gaussian distance weights for the distance between $i$-$j$ and $i$-$k$, respectively. A visualization of this step can be seen in Fig. 1. Visually, note that as opposed to the MH3SFCA method introduced by Jörg et al. [31], no subzones are used in our version of the method. Instead, each reachable pair of supply and demand location is assigned an individual driving time and thus an individual weight.

**Fig. 2:**
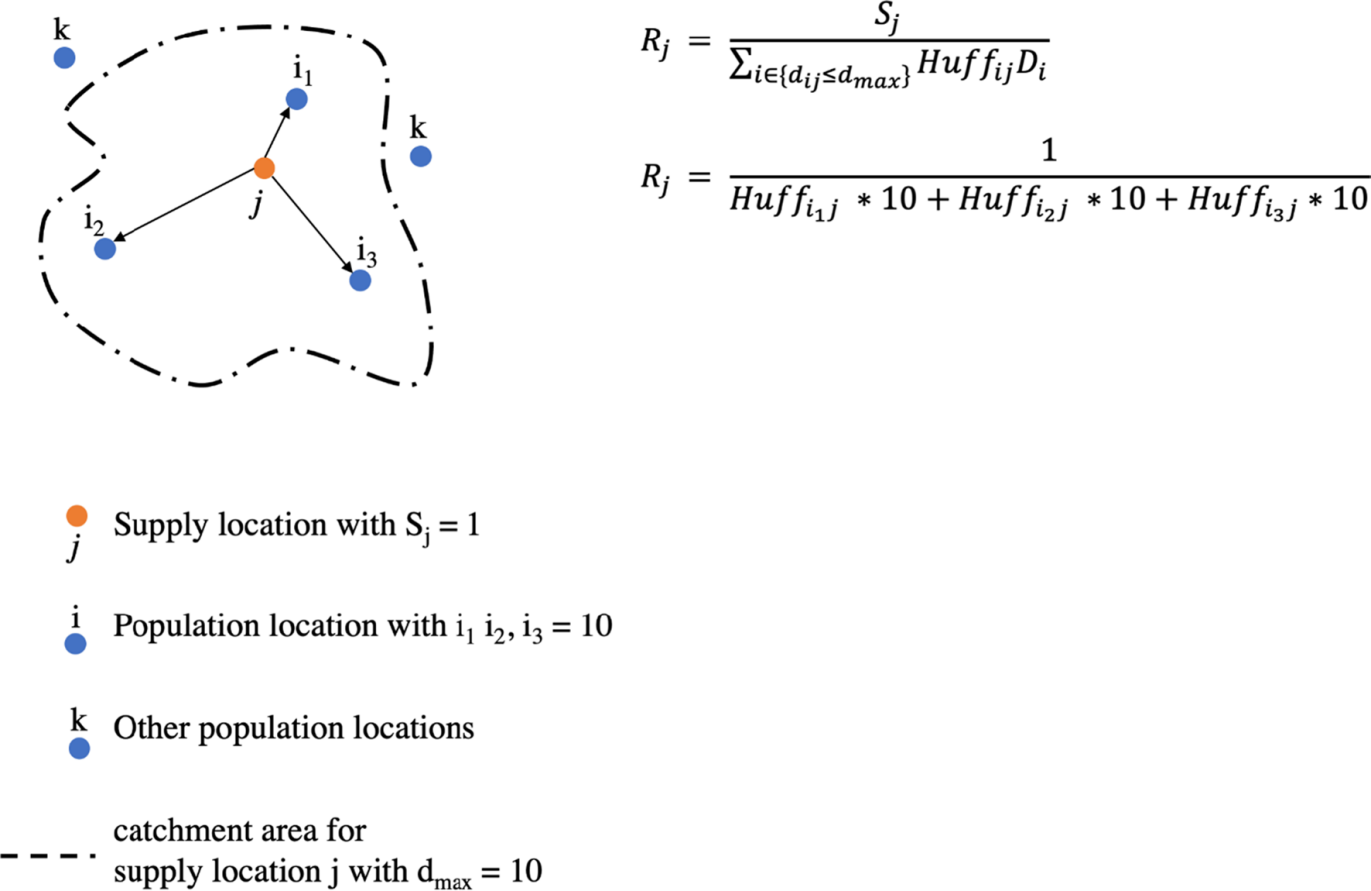
The second step of the MH3SFCA method. The supply–demand-ratio $R_{j}$ is calculated by dividing the capacity of $j$ by the sum of the products of the $Huff_{ij}$ values and the number of inhabitants for each reachable demand location $i_{1}$, $i_{2}$ and $i_{3}$

*Step 2*: In the second step, the supply–demand-ratio $R_{j}$ is calculated by

$$R_{j } = \frac{{S_{j} }}{{\mathop \sum \nolimits_{{i \in \left\{ {d_{ij} \le d_{max} } \right\}}}^{{}} Huff_{ij} D_{i} }}$$

where $D_{i}$ denotes the demand for each population location $i$ located within the catchment of $j$ ($d_{ij} \le d_{max}$). A visualization of this step is depicted in Fig. 2.

*Step 3*: Lastly, in the third step, the spatial accessibility index $A_{i}$ is calculated for each population $i$ by

$$A_{i } = \mathop \sum \limits_{{j \in \left\{ {d_{ij} \le d_{max} } \right\}}}^{{}} Huff_{ij} R_{j} W_{ij}$$

**Fig. 3:**
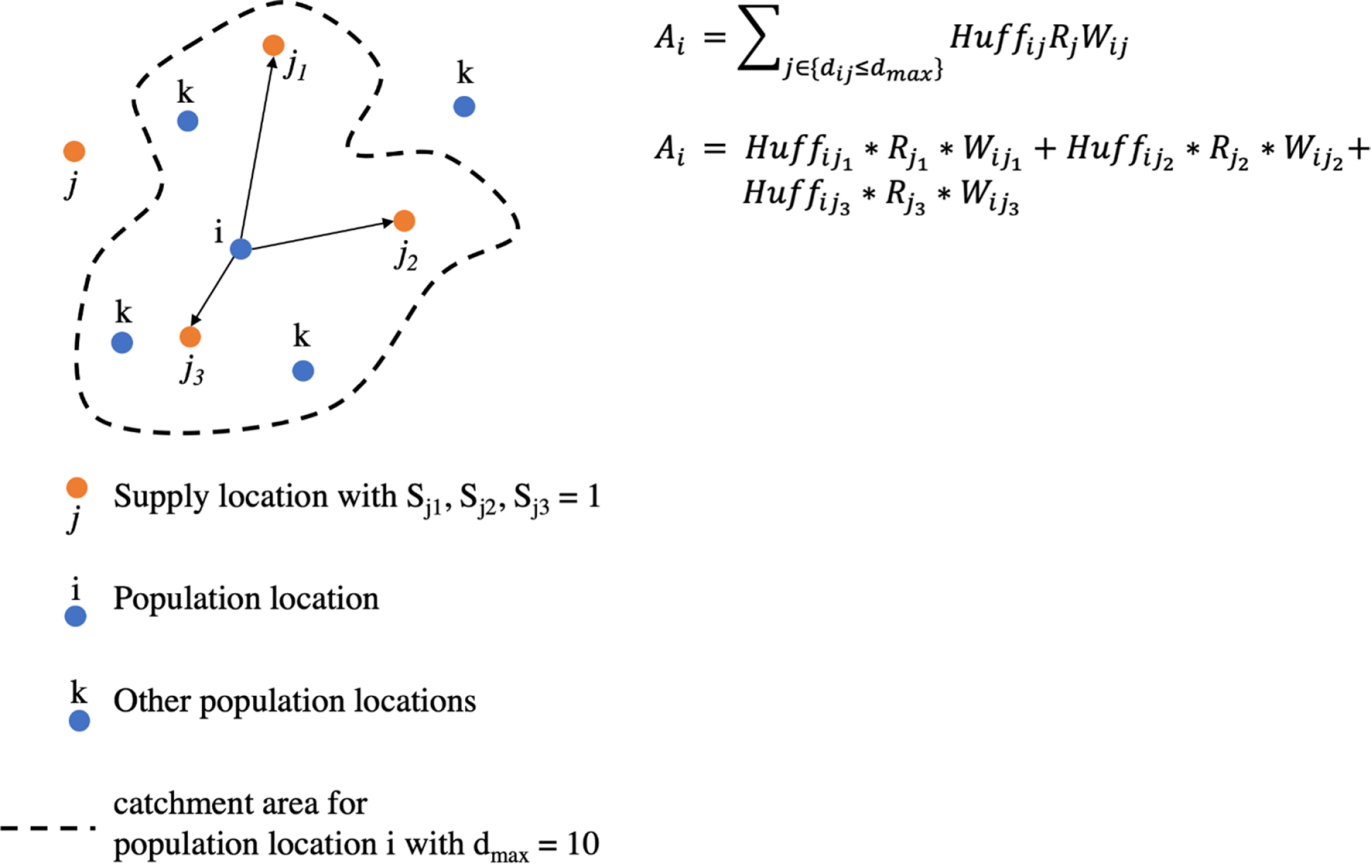
The third step of the MH3SFCA method. The accessibility index $A_{i}$ is computed by summing up the products of the $Huff_{ij}$, $R_{j}$ and $W_{ij}$ values for the reachable supply locations $j$. As no subzones are used in this modified version, the weights are the individual weights

Figure 3 is depicting a visualization of the third step.

## Results: application of the MH3SFCA method to a test dataset

**Fig. 4:**
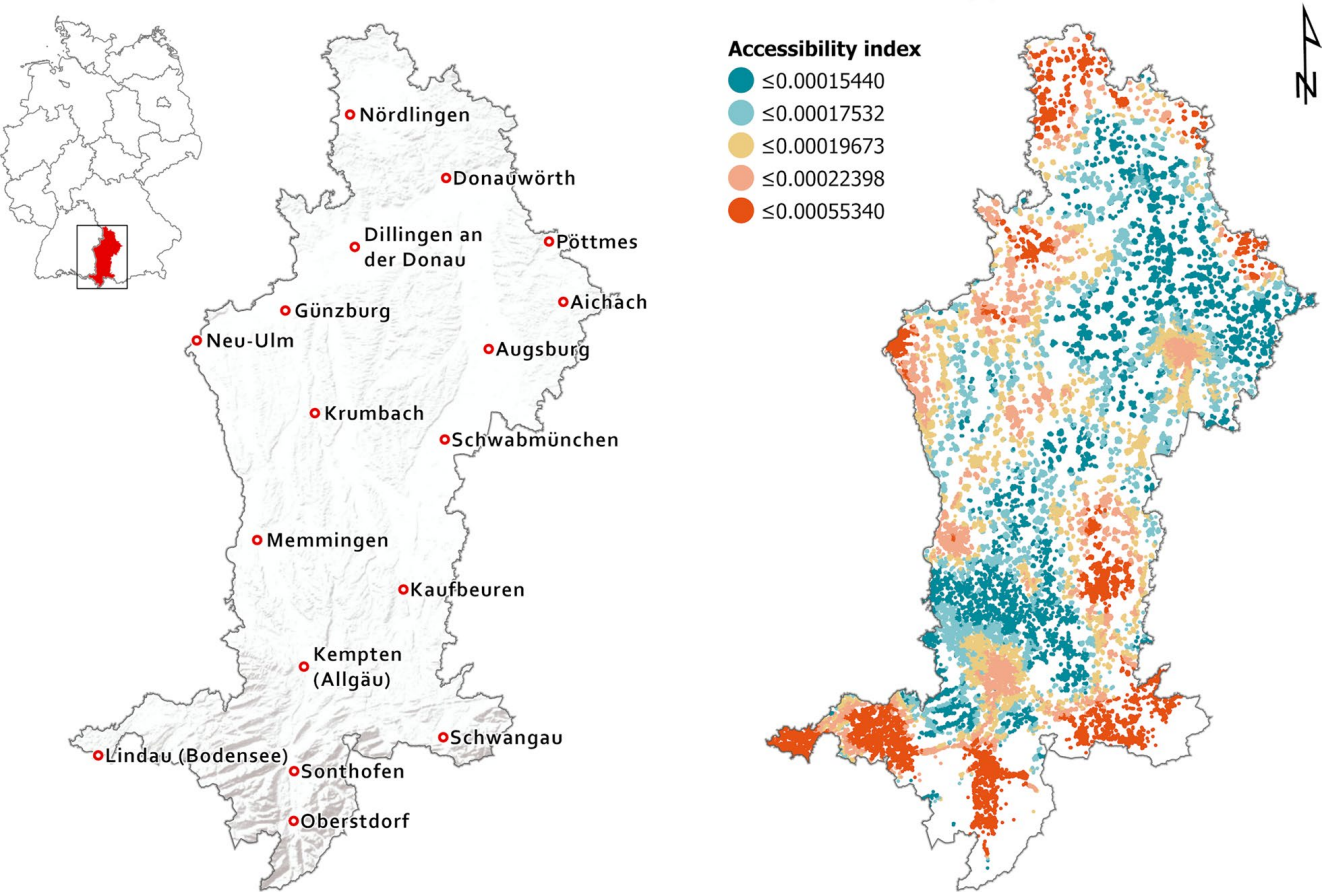
Maps featuring the position and an overview of Swabia (left) and the results of the application of the MH3SFCA method on the case study’s dataset (right). High values of the accessibility index are portrayed by (dark) red, average values by yellow and low values by (dark) blue

The initial result of this case study is a point layer indicating the accessibility index of GPs. It comprises the accessibility indices for 83,418 population locations in total. Generally, retaining the results on their original scale provides the advantage that large-scale patterns can be detected. The results of the MH3SFCA method for the datasets of the case study’s area, Swabia, are presented in Fig. 4. In order to visualize the results, various possibilities have been tested. Initially, the raw results consisting of the hectare grid cell centroids were pictured using a color range corresponding to the values of the accessibility indices. Alternatively, the accessibility indices were joined back to the original 100 by 100 m grid cells and visualized again by using a color range. However, the latter method was not useful to show the results within Swabia as the legibility was severely impaired due to the small scale of the grid cells. Therefore, the grid cell centroids were used and several blank spaces remain in the map as there is no population due to forests, agriculture or similar land use.

In the map, the accessibility indices are truncated to eight decimals. We choose quantiles as the classification method because it allows for comparing the tendencies of the index values. On average, there are about 0.0002 GPs available for each person in Swabia. It should be noted at this point that this average value is however influenced by the Gaussian weights which are associated to the maximum travel time as well as by the coefficient of friction $\beta$. The minimum values show that there are no population locations that received an accessibility index of zero. Thus, all of the 83,418 population locations in the results do have access to one or more GPs. In other words, there are no locations that needed more than 30 min to reach a GP in this exemplary test dataset.

Generally, spatial accessibility indices are distributed in an inhomogeneous as well as polycentric pattern. The differences in spatial accessibility are found mainly between urban and rural areas. While cities such as Augsburg, Neu-Ulm and Kaufbeuren, and particularly their city centers, exhibited above-average values of accessibility, more rural areas such as the territories around Kempten or the larger surrounding area of Augsburg are assigned below-average values. Moreover, spatial accessibility is above average in the southernmost part of Swabia around Lindau, Oberstdorf and Schwangau. Additionally, for the direct surrounding areas of larger cities, smaller cities and towns, mostly average to slightly above-average accessibility indices are calculated. This is for instance the case for Augsburg, Kaufbeuren or Kempten. Eventually, the transitions from lower to higher values of accessibility or vice versa in general are smooth rather than abrupt. On closer inspection, the accessibility indices within the Augsburg area only comprise the light red and yellow quintile. Hence, there is no very high accessibility (dark red) in this area. The same applies to the areas of Kempten and Memmingen. In contrast, the accessibility indices are particularly high in the region to the east of Lindau, the northernmost part of Swabia around Nördlingen, Dillingen an der Donau, Günzburg and Kaufbeuren. Lastly, it is noteworthy that for the Donauwörth area, only below-average (blue) values of accessibility come up.

### On a larger scale—exemplary analysis for Augsburg based on the test dataset

**Fig. 5:**
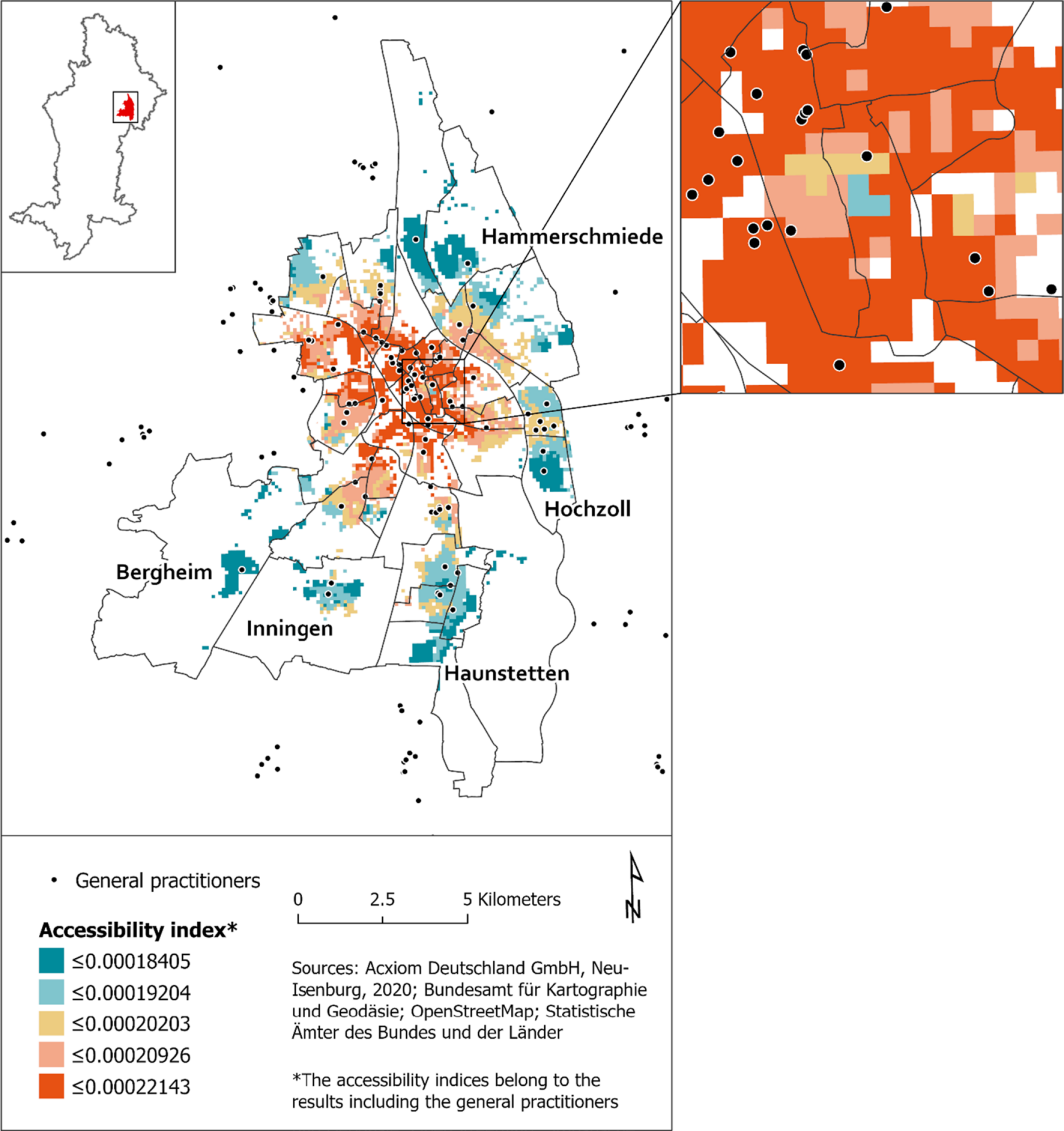
The position of Augsburg within Swabia (top left) and the resulting accessibility indices for the general practitioners within the city of Augsburg based on the test dataset. The red colors indicate high values of the index while the blue colors indicate low values

To show the spatial access to GPs on a larger scale, the city of Augsburg is used as a test area. Augsburg has about 300,000 residents. With this population size, Augsburg is Swabia’s largest and Bavaria’s third largest city. Thus, it is representative for areas characterized by urban structures. As before, the accessibility indices within the area are classified using the classification method quantiles. Note that the absolute values of the indices differ from the ones presented in Fig. 4. In the map below (Fig. 5), the values of the accessibility index within Augsburg are featured within the 100 by 100 m grid cells. Additionally, the practices of GPs are depicted by the black points.

The resulting values ranged from 0.000118 (min) to 0.000221 (max). As apparent from the map, the highest accessibility can be enjoyed in the densely populated areas in the city center where most GPs gather. Moving away from the city center, the values gradually decline. In the areas surrounding the city center, high to average values are prevalent. Lower values are found in the surrounding communities such as Hammerschmiede, Hochzoll, Haunstetten, Inningen and Bergheim. In addition, a fine-scale pattern is revealed within the city center of Augsburg. This pattern is magnified in the map frame on the top right. In the pattern, below average values (light blue) are surrounded by average and above-average values of accessibility.

## Discussion of the results

As we base our analysis on exemplary test data, the initial findings concerning the spatial accessibility to GPs have only limited validity. Generally, the rural areas of Swabia exhibited lower values of accessibility in comparison to the urban areas. Moreover, the distribution of accessibility complied with a polycentric pattern as the higher values of accessibility are located in multiple urban areas. These findings are well in accordance with those of comparable German studies (see e.g. [49–51]) as well as studies from other areas (see e.g. [10, 31, 34, 41]). A reason for the heterogeneity between urban and rural areas are for instance longer travel times to the nearest doctors for the inhabitants in rural areas. Another reason may be lower supply capacities in rural areas and correspondingly higher capacities in urban areas.

Our case study indicates that the MH3SFCA method can help to find various differences in accessibility. There might be a need to assess these differences more thoroughly as their origin is not always clear. For example, the reason for the low values of accessibility in and around Donauwörth might be a sparse distribution of GPs in this area combined with a rather high number of inhabitants in the city of Donauwörth.

Another finding that requires closer analysis are the remarkably high values of accessibility in the south of Swabia. The reason for these values, as a comparable study by Jörg et al. [31] suggests, is likely the high amount of summer and winter tourism in these areas. Holetschek [52] also emphasizes that traditions in the healthcare sector have a long history in the alpine regions. He moreover stresses the high number of different healthcare establishments in this area. The combination of these factors may lead to the calculation of high accessibility indices: The rather low number of residents in these areas are assigned high capacities of the various surrounding supplies. However, the MH3SFCA method does not consider tourism or – more specifically – overnight stays. As a result, the demand in these southern regions may well be underestimated because even if there is a large supply capacity, it is most likely not fully allocated to the actual residents in these areas. Instead, a high number of tourists might access these supply capacities.

On a larger scale, the city center of Augsburg shows higher values of accessibility in comparison to the surrounding suburban districts. This can be anticipated as there is a noticeably higher density of GPs within the city center. However, three population locations in the city center have an accessibility index that is notably lower than the index of the surrounding population locations. A closer look at the area in question shows that the most likely reason for these low values is the difference in travel time to GPs as the three population locations with lower accessibility are located near one-way streets. Indeed, when the mean travel times of the three locations are compared to the surrounding population locations, the three locations have a higher mean travel time to GPs than the surrounding locations. Furthermore, the accessibility gradually increases when moving further north from the three population locations with lower access (see Fig. 5). This might be due to the influence of the one-way streets as the travel time decreases when moving further away from these streets.

These findings indicate that the MH3SFCA method can be used to detect differences in accessibility on a larger scale. Considering the high probability that the differences in accessibility are caused because of differing travel times, such a pattern could not have been found by using a simpler method such as a supply–demand-ratio.

### Strengths and weaknesses

Access to healthcare is a fundamental human right and this includes access to basic and acute healthcare, which is fundamental to achieving the United Nations’ health for all goals. Poor access to healthcare services causes poor health. Evidence demonstrates that even in high and upper middle-income countries rural areas are underserved due to poor(er) education, lack of economic resources, transportation systems and inadequate medical resources [7]. Our paper serves as a point of reference for spatial understanding of accessibility to healthcare services and can be applied as such in further research. The improved Huff model, incorporating a more realistic way to model the spatial distance decay effect, can be implemented in predicting and planning for better access and thus better health outcomes in all settings.

Considering the emerging global health threats, such as aging society, aging and lack of health workforce, climatic and economic burdens; stakeholders shaping the health programs, distributing finances and making executive decisions in terms of priorities need realistic and precise models (naturally based on accurate data) for global, country-level and regional policy making. Studying the spatial dimension, such as the MH3SFCA method, are of utmost importance to make sustainable decisions regarding accessibility of healthcare services. Accordingly, our case study demonstrates that the number of GPs is highest in densely populated areas, such as in the city center. Moving away from the city center, the values gradually decline. Application of MH3SFCA helps to identify the underserved areas, which can be included in cross-sectional planning to remove the existing barriers.

This is the first academic paper introducing the MH3SFCA method to international audience. Strengths of the MH3SFCA method comprise the possibility to assess accessibility to GPs on different scales. Furthermore, different regions as well as suppliers of different kinds can be compared to one another. Due to this characteristic, the method would be fitting to compare spatial aspects of accessibility as well as availability to enhance decision-making and planning [51].

Limitations of the method are the difficulty when interpreting why certain patterns or outcomes occur in the results as the method takes into account a high number of factors in comparison to other methods that measure spatial accessibility. Due to uncertainties in the data, the resulting indices are limited. They reflect the results based on the input data. Further limitations involve choices upon certain parameter values in the MH3SFCA method, namely the travel time threshold, the coefficient of friction $\beta$ in the Gaussian function and the choice upon a certain distance decay function in itself. Population and population density is highly dynamic [53]. As we assume people sleep at home, we consider our population data to be at a night-time state. However, people might rather access healthcare for example on their way to or from work [54]. Therefore, the input data for the population might be changed dynamically to represent different starting locations.

## Conclusion and implications for future practice

The main contribution of this study is an innovative and new approach to calculate the already existing MH3SFCA method by Jörg et al. [31]. Instead of using a binary or subzone-based approach however, we use individual Gaussian weights to represent individual driving times from the population to the healthcare sites. The application of the MH3SFCA method on large-scale data on the one hand provides an overview of accessibility for the whole study area. On the other hand, the results can additionally be assessed at larger scales such as on the level of single municipalities. As a result, larger-scale variations in spatial accessibility can be investigated.

The results indicate that the MH3SFCA method is suited for comparing the accessibility of different regions. Due to this characteristic, the method would be fitting to compare spatial aspects of accessibility as well as availability to enhance decision-making and planning [51]. Depending on the objective, it might be necessary to further analyse the results of the method. As a lot of factors have to be taken into account, the outcomes are too complex for a direct and clear interpretation of why indices are rather low or high. This characteristic also reflects the method’s closeness to reality since accessibility is a complex topic. Albeit the results of the MH3SFCA method can be used to indicate over- and undersupplied areas, the absolute values of the indices do not inherently define accessibility to be too low or too high. Instead, the indices compare the spatial relationships between each supply and demand location. As a result, the higher the value of the accessibility indices, the higher the opportunities for the respective population locations.

To effectively detect over- or undersupply, either further analysis must be conducted or different (legal) constraints must be applied. For instance, Voigtländer and Deiters [55] set a time limit of 30 min to the nearest provider. This is a reasonable measure. The BPL-RL suggests certain ratios of supply to demand are required to fulfil the conditions of either over- or undersupply. The MH3SFCA method might help to investigate the setting as well as adjustment of such ratios. Additionally, by the inclusion of various relevant factors such as supply capacity, demand and a continuous approach to distance decay, the method represents a more sophisticated approach compared to simple supply–demand-ratios.

The FCA methods in general as well as the MH3SFCA method have various advantages over alternative and particularly simpler methods. However, the way in which the method was used in this paper also led to a number of shortcomings, which include:

- The case study is utilizing a subset of test data. Therefore, the results are not valid in terms of planning purposes. These results show the possibilities of utilizing the innovative modified MH3SFCA methodology.
- The case study only employs the travel mode by car. However, in some cases it might be appropriate to employ other modes of transport. This is especially important when assessing the accessibility of specific populations, who might prefer a different transport mode.
- Another shortcoming of the case study is the omission of the edge effect. Normally, people will travel across administrative borders (apart from country borders) to access healthcare. The ability to include this edge effect is an advantage of the FCA methods over more traditional methods such as simple supply–demand-ratios. Therefore, additional data about healthcare providers and populations stemming from a buffer zone around the study area should also be included to account for the edge effect [19, 44, 54, 56]. Therefore, a possible inaccuracy due to the missing inclusion of edge effects should be kept in mind particularly when interpreting the accessibility indices near the border in this case study.
- The computing times of the MH3SFCA method might also be seen as a possible limitation. Especially for larger areas and when employing large-scale data (such as in this study), the computation time might be considerably high. For example, with the hardware that was used in this study, the calculation for 11,291,166 pairs of GPs and population locations took 12 h 32 min.
- Since it is not known to what degree the state and locations of healthcare as well as the condition of the OSM network in Swabia is transferable to other administrative regions within Bavaria and especially to other federal states, generalizing the findings of this study to other areas in Germany should not be done without further analysis.

Regarding the transferability of the MH3SFCA method, various possibilities exist. First, it may be applied to other areas in Germany, the European Union or different countries. The application of the method does require high quality data to avoid a “garbage in, garbage out” situation. Furthermore, the method may be applied to alternative providers of healthcare like hospitals, pharmacies or gynecologists. It is also possible to use the method to assess access to other fields of interest such as jobs, education or food.

## Data Availability

The most current basis for the network data used in this study is publicly available at https://download.geofabrik.de/europe/germany/bayern/schwaben.html. The population locations are publicly available at https://www.zensus2011.de/DE/Home/Aktuelles/DemografischeGrunddaten.html. The data comprising the locations of the general practitioners has been provided only for the use in this study by Acxiom Deutschland GmbH. They provide information on conditions for access. https://www.acxiom.com/.

## Abbreviations

COVID-19: Coronavirus disease 2019
BPL-RL: Bedarfsplanungs-Richtlinie
EU: European Union
FCA: Floating catchment area
GIS: Geographic Information System
GPs: General practitioners
MH3SFCA: Modified Huff Model three-step floating catchment area
NCDs: Non-communicative diseases
OECD: Organisation for Economic Cooperation and Development
UN: United Nations

## Footnotes

1 https://www.zensus2011.de/DE/Home/Aktuelles/DemografischeGrunddaten.html, accessed on March 7, 2020.

2 https://qualitysafety.bmj.com/content/qhc/early/2015/09/10/bmjqs-2015-004411.full.pdf, accessed on June 21, 2020.
